# A Multi-Objective Framework for Biomethanol Process Integration in Sugarcane Biorefineries Under a Multiperiod MILP Superstructure

**DOI:** 10.3390/e27111162

**Published:** 2025-11-15

**Authors:** Victor Fernandes Garcia, Reynaldo Palacios-Bereche, Adriano Viana Ensinas

**Affiliations:** 1 Center of Engineering, Modeling and Social Science Applied, Federal University of ABC, Santo André 09280, Brazil; v.garcia@ufabc.edu.be (V.F.G.); reynaldo.palacios@ufabc.edu.br (R.P.-B.); 2 Department of Engineering, Federal University of Lavras, Lavras 37200, Brazil

**Keywords:** sugarcane biorefinery, biomethanol production, multi-objective optimization, MILP-based superstructure, heat integration

## Abstract

The growing demand for renewable energy positions biorefineries as key to enhancing biofuel competitiveness. This study proposes a novel MILP superstructure integrating resource seasonality, process selection, and heat integration to optimize biomethanol production in a sugarcane biorefinery. A multi-objective optimization balancing net present value (NPV) and avoided CO_2_ emissions reveals that energy integration improves environmental performance with limited economic impact. The model estimates the production of up to 66.85 kg of biomethanol/ton sugarcane from bagasse gasification, 40.7 kg e-methanol/ton sugarcane via CO_2_ hydrogenation, and 3.68 kg of biomethane/ton sugarcane from biogas upgrading. Hydrogen production through biomethane reforming and photovoltaic-powered electrolysis increases methanol output without raising emissions. The integrated system achieves energy efficiencies of up to 57.3% and enables the avoidance of up to 493 kg of CO_2_/ton sugarcane over the planning horizon. When thermal integration is excluded, efficiency drops by 8% and net energy production per area falls by 11%, due to the need to divert bagasse to cogeneration. Although economic challenges remain, CO_2_ remuneration ranging from USD 3.27 to USD 129.79 per ton could ensure project viability. These findings highlight the role of integrated energy systems in enabling sustainable and economically feasible sugarcane biorefineries.

## 1. Introduction

In recent decades, as environmental concerns have grown, biofuels have come to play a key role in building a low-carbon economy [1]. In this scenario, Brazil stands out as one of the largest global producers of biofuels. Driven by strategic public policies, the country is one of the leaders in the production and use of ethanol and biodiesel, which play a central role in its energy matrix [2,3]. Despite these advances, the biofuels sector faces strategic challenges. Biodiesel, the majority of whose production in Brazil is based on the transesterification of vegetable oils, is critically dependent on methanol as a reagent, an input that is not produced in Brazil, exposing the production chain to price fluctuations and logistical vulnerabilities, which can impact the sector’s competitiveness and security [4]. Given the importance of the sugar-energy sector to the Brazilian economy, it is strategic to explore the integration of biomethanol production from sugarcane biomass, which is emerging as a promising alternative to overcome this dependence on imported inputs. As well as aligning biofuel production with the principles of the circular economy, this is also a way of diversifying products and maximizing the value of sugarcane, so that the sector is increasingly aligned with the concept of biorefineries [5].

A biorefinery can be defined as a set of industrial processes which, in an integrated way, can transform a given biomass into different marketable products. By exploiting and taking advantage of the synergies between the different processes, it is possible to optimize production and the use of existing resources [5]. Despite their importance, the development and expansion of biorefineries still need to overcome some challenges such as high initial investments, which can result in a lack of competitiveness of biofuels [6]. Thus, identifying the most appropriate configuration for these industrial complexes is fundamental to optimizing their economic performance.

The use of methods based on mixed integer linear programming (MILP) is an alternative adopted by different studies to identify synergies between processes and optimize their economic performance [7,8,9]. However, the quest to maximize profit can lead to choices that compromise the biorefinery’s environmental performance. It is therefore essential to find a balance between profitability and environmental performance, as well as to understand the impact that certain technologies can have on the system. To deal with problems of this nature, multi-objective optimization is an alternative capable of providing the information needed to understand the impact of improving one indicator on the other indicator [10,11]. When used in the context of economic and environmental optimization, it can identify a set of alternative solutions that balance environmental and economic performance, which are called optimal solutions and form the so-called Pareto curve. The Pareto curve is made up of solutions that are better in at least one criterion, without being worse in any other criterion, compared to all the other possible solutions. By analyzing these solutions, it is possible to understand the trade-offs between the existing alternatives, allowing the decision maker to choose the most appropriate solution based on preferences and operational or strategic constraints [11].

The applicability of multi-objective optimization in the context of biorefinery development can be seen in different studies [12,13,14]. Medeiros et al. [15] optimized the production of ethanol from biomass waste (sugarcane bagasse and eucalyptus) using a gasification and syngas fermentation process. The study evaluated the impact of process variables on three objectives: minimum selling price, energy efficiency and carbon footprint. The main conclusion was that this technology is not yet economically competitive with first-generation ethanol. In order to reduce the production costs of second-generation ethanol, Kamzon et al. [16] conducted a study to optimize the pretreatment of Moroccan sugarcane bagasse with dilute sulfuric acid. The main objective was to maximize the production of fermentable sugars while minimizing the formation of inhibitors. To balance these two objectives, the authors employed a desirability function, achieving a remarkable result of 92%. To account for the economic, environmental and social dimensions in the design and planning of an international sugarcane supply chain network for biofuel, Gilani and Sahebi [17] developed a robust multi-objective mixed integer linear programming (MILP) model that considers profit maximization, job creation, and minimization of environmental impacts.

The integration of methanol production into sugarcane biorefineries remains a complex challenge, and most studies still rely on sequential optimization approaches or static configuration analyses, which hinder process scalability and prevent the full exploitation of synergies across production pathways, often resulting in high computational costs and suboptimal global performance. Moreover, although previous works have proposed methodologies for biorefinery design, to the best of our knowledge, no study to date has simultaneously accounted for key elements such as resource seasonality, process selection and scaling, heat integration across cascades, and utility system design within a single unified framework. To address these gaps, the objective of this study is to present a multi-objective MILP-based superstructure capable of representing (i) temporal variability of resource availability, (ii) process selection and scale adjustment, and (iii) simultaneous heat integration and utility definition to analyze the trade-offs between economic (NPV) and environmental (total CO_2_eq avoided) outcomes resulting from the incorporation of emerging technologies for biomethanol production in the biorefinery.

## 2. Materials and Methods

**Figure 1:**
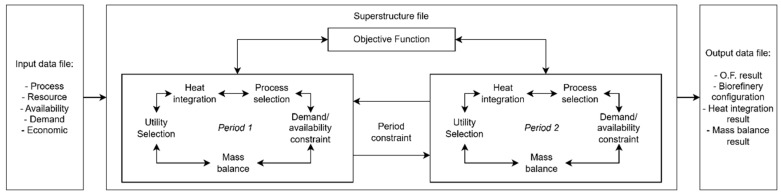
Schematic representation of the superstructure’s information flow.

The formulation presented in this work deals with an extension and adaptation of previous work performed by the authors [18,19]. Previously, the restrictions considered global mass and energy balances, the aim of which was to select and adjust the scale of the processes that make up the biorefinery, as well as heat integration between them and utility selection. However, seasonality of resources was not taken into account. Compared to previous versions, the methodology described in this article stands out for incorporating mathematical restrictions related to multi-period and multi-objective optimization. The inclusion of time constraints aims to capture the impact of the seasonality of resources on the development of biorefineries. To identify solutions that do not only consider the best performance of a single aspect of the biorefinery, a multi-objective optimization method with two conflicting objective functions was used. The current formulation uses an iterative method to generate the Pareto curve, allowing different solutions to be explored that balance the two objectives considered: environmental and economic. Surrogate models, based on previously published papers, were used to represent each process, its main thermal streams, and the resources consumed and produced. The constraints were modeled as a Mixed Integer Linear Programming (MILP: Mixed Integer Linear Programming) model and implemented in the LINGO program [20]. Figure 1 shows a representation of how the formulation presented in this paper works.

In the following subsections of this section, the formulation of the optimization problem is presented and explained. Initially, the sets and subsets that make up the superstructure are presented, as well as their relationships and elements. Then, the constraints used in the formulation are presented and how they work. In Section 3, an explanation of the case study, the main processes and parameter values considered, and the literature references used are provided. In Section 4, the results are provided and explained, and finally, in Section 5, the conclusions are presented.

### 2.1. Optimization Problem Formulation

#### 2.1.1. Main Sets and Subsets

The optimization problem is organized into different sets and subsets, with each set responsible for storing distinct elements. The three main sets are RESOURCE (R), UNIT (U), and PERIOD (PE). Each element, designated by r (r belonging to R), signifies a resource that can be converted into another resource, such as cane sugar, electricity, or carbon dioxide. The elements u (u ∈ U) are representative of the processes that can be incorporated into a biorefinery, such as a cane sugar factory or a cogeneration system. The elements contained in PE (pe ∈ PE) represent the distinct periods that comprise a year.

The elements in the PROCESS subset (PU|PU ⊂ U) represent the process that has at least one thermal stream. In this sense, every element in pu (pu ∈ PU) is considered in the heat integration constraints. The UTILITIES (UT|UT ⊂ R) subset stores the elements that represent thermal utilities, such as steam and cold water, that are considered in the heat integration. Hot and cold utilities are segregated into two UTILITIES subsets, HUT (HUT ⊂ UT) and CUT (CUT ⊂ UT), respectively. Another subset of RESOURCE is STORAGE (ST ⊂ R), whose elements are the resources that can be stored.

The elements contained within the subset PROCESS (PU|PU ⊂ U) are defined as processes that possess at least one thermal stream requiring adjustment of temperature. Therefore, all elements pu (pu ∈ PU) are considered in the context of energy integration. The subcollection UTILITIES (UT|UT ⊂ R) contains the elements that represent the thermal utilities, such as high- and low-pressure steam, which are considered in the context of energy integration. The hot and cold utilities are divided into two distinct UTILITIES (UT) subsets: HUT (HUT ⊂ UT), and CUT (CUT ⊂ UT), respectively. Another RESOURCE subset, named STORAGE (ST ⊂ R), encompasses the resources that are amenable to storage.

#### 2.1.2. Process Selection and Scale Adjustment

Depending on the context evaluated, certain technologies may or may not be part of the biorefinery. Therefore, to leave the choice of processes that will be present in the biorefinery up to the optimization problem, constraint (1) was used. In this constraint, ${CapMin}_{u}$ and ${CapMax}_{u}$ represent the maximum and minimum scale adjustment values that a given process can have. $y_{u}$ is a binary variable that takes on the value 1 if unit u will be present in the biorefinery, or 0 if it is not present.

(1) $${CapMin}_{u}{\;y}_{u}\;<\;w_{u,\mathrm{pe}}\;\leq \;{CapMax}_{u}y_{u}\;\;\;\;\;\;\;\;\forall u\in U,\forall pe\in PE$$

As in previous studies, each process within the superstructure is represented by a surrogate model, with a defined reference scale, its key flows of production and consumption of resources, and its investment. To adjust the scale of a process for each context evaluated, a continuous scale adjustment multiplier variable (w_u,pe_) was developed. To adjust flow rates linearly and proportionally, it is necessary to multiply this variable by the parameters of consumption $\left({IAR}_{u,r}\right)$ and production $\left({OAR}_{u,r}\right)$ of resources. To illustrate, in instances where a given process requires operation at twice its reference capacity, the value of the scaling variable would be equivalent to two. Conversely, if the process demands operation at half its reference capacity, the value of the scaling variable would be reduced to half. It is important to note that the same unit can exhibit different values of w_u,pe_ in different periods. A unit is capable of operating at different times of the period, thus allowing it to adjust its operation according to the seasonality and availability of a resource at a given time of the year.

#### 2.1.3. Mass Balance Constraints

Given that the superstructure considers the acquisition, sale, consumption, production and storage of resources, when carrying out the mass balance, it is important to consider all the possible flows that a given resource may present. However, not all resources are subject to the same flow, as is the case with thermal utilities, which cannot be stored. Therefore, depending on the resource, it is necessary for its mass balance to consider or disregard certain flows. For resources that cannot be stored and are not utilities (r∈R−(ST∪UT)), the mass balance is given by the Equation (2). The variables ${bought}_{r,pe}$ and ${sold}_{r,pe}$, represent the mass bought and sold of resource r and period pe, respectively. The variables ${prod}_{u,r,pe}$ and ${cons}_{u,r,pe}$ represent the mass produced and consumed, respectively, by unit u, of resource r and period pe, and are determined by the Equations (3) and (4), respectively. ${fop}_{pe}$ represents the duration in hours of the period pe.

(2) $${bought}_{r,pe}+\sum_{u}{prod}_{u,r,pe}={sold}_{r,pe}+\sum_{u}{cons}_{u,r,pe}$$

(3) $${OAR}_{u,r}.W_{u,pe}.{fop}_{pe}-{prod}_{r,upe}=0$$

(4) $${IAR}_{u,r}.W_{u,pe}.{fop}_{pe}-{cons}_{r,u,pe}=0$$

For thermal utilities, the mass balance is given by Equation (5). It is important to highlight the consideration that thermal utilities cannot be stored. As in previous work, the superstructure carries out utility selection simultaneously with energy integration and scale adjustments, so that the consumption of a utility by a process cannot be given by a parameter, but rather by a variable. Thus, for thermal utilities, Equation (4) needs to be rewritten in the form of Equation (6).

(5) $$\sum_{u}{prod}_{u,r,pe}=\sum_{u}{cons}_{u,r,pe}$$

(6) $${massUtility}_{u,r,pe}.{fop}_{pe}-{cons}_{u,r,pe}=0$$

For resources that can be stored, the material balance needs to consider the quantity stocked of that resource. So, for these resources, the material balance is carried out using Equation (7), where ${stockIn}_{r,pe}$ and ${stockOut}_{r,pe}$ represent the mass flow of resource r entering and leaving stock, respectively, at period pe.

(7) $${bought}_{r,pe}+\sum_{u}{prod}_{u,r,pe}+{stockOut}_{r,pe}={sold}_{r,pe}+\sum_{u}{cons}_{u,r,pe}+{stockIn}_{r,pe}$$

#### 2.1.4. Stock Mass Balance

**Figure 2:**
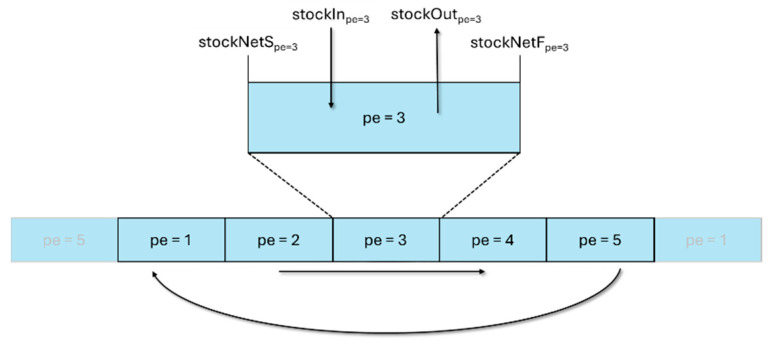
Schematic representation of stock flows over a period.

Given that the stock mass of a stock resource can be consumed in different periods, it is necessary to carry out a material balance of each stock resource. To achieve this, Equation (8) was used. The stock balance considers the balance at the beginning and end of the period, which are accounted for by the variables ${stockNetS}_{r,pe}\;$ and ${stockNetF}_{r,pe}$, respectively. The variation between them is the difference between the total quantity of the resource that entered stock in that period (${stockIn}_{r,pe}$) and the total quantity of the resource that left in that period (${stockOut}_{r,pe}$). The optimization problem developed considers that the initial stock at the start of each period is equal to zero (${stockNetS}_{r,pe=1}=0$), as well as considering that the balance at the start of a period is equal to the balance at the end of the previous period (${stockNetS}_{r,pe}=\;{stockNetF}_{r,pe-1}$), as shown in Figure 2

.
(8) $${stockNetF}_{r,pe}={stockNetS}_{r,pe}+{stockIn}_{r,pe}-{stockOut}_{r,pe}$$

#### 2.1.5. Availability and Demand Constraints

For a given resource to be purchased before it is consumed, it must be available in that period. To ensure that the total purchased of that resource does not exceed its annual availability, the following equation was used (9). Equations (10) and (11) guarantee that the quantity of a resource purchased in a period is limited to its availability in that period. The binary parameter ${yAvail}_{r,pe}$ has a value of one for cases in which resource r may be available in period pe, or has a value of zero when resource r is not available in that period. This parameter was introduced to limit the availability of resources in different periods. For example, suppose that a certain resource can only be available in the pe period equal to 1 and 2, but cannot be available in the period equal to 3 for some known or unknown reason, this parameter limits the purchase of this resource to periods 1 and 2, leaving it unable to be purchased in period 3. The optimization problem considers that in the first period of the cycle, the availability of a certain resource is equal to its annual availability, as shown in Equation (12).

(9) $${avail}_{r}\geq \sum_{pe}{bought}_{r,pe}$$

(10) $${availPeriod}_{r,pe}={availPeriod}_{r,pe-1}-{bought}_{r,pe-1}$$

(11) $${availPeriod}_{r,pe}.{yAvail}_{r,pe}\geq {bought}_{r,pe}$$

(12) $${availPeriod}_{r,pe=1}={avail}_{r}$$

In order to limit the quantity sold of a resource to the demand for that resource in that period, the following equation was used (13), where ${demandP}_{r,pe}$ is a parameter that represents the demand value of resource r, at period pe.

(13) $${demandP}_{r,pe}\geq {sold}_{r,pe}$$

#### 2.1.6. Heat Integration and Utilities Selection

To consider heat transfer between processes, energy balance constraints and energy integration between cascades were used, based on the work of Bagajewicz and Rodera [21] and adapted from the work of Garcia and Ensinas [18] in order to take the multi-period aspect into account. As input data, the superstructure needs to receive the number of stages (s), as well as the inlet (Te_s_) and outlet (Ts_s_) temperature values for each of them. It is also necessary to provide the minimum hot (MER_pu_) and cold (UF_pu_) utility consumption for the reference scale of each pu element, as well as the input temperature (Tin_pu,n_), output temperature (Tout_pu,n_), and heat capacity (MCp_pu,n_) values for each thermal current n. Equation (14) guarantees that the heat demand of a process will be met by a hot utility (Qu_pu,ut_) or transferred from other processes (Qin_pu_). Similarly, Equation (15) guarantees that all excess heat from a process will be removed by a thermal utility or transferred to another process (Qout_pu_). The consumption of a hot utility ut by a pu process is determined by Equations (16)–(18), which identify the stages that demand heat and limit the quantity supplied by the amount demanded.

(14) $${\mathrm{MER}}_{\mathrm{pu}}w_{\mathrm{pu},\mathrm{pe}}=\sum_{\mathrm{ut}}{\mathrm{Qu}}_{\mathrm{pu},\mathrm{ut},\mathrm{pe}}+{\mathrm{Qin}}_{\mathrm{pu},\mathrm{pe}}\;\;\;\;\;\;\;\;\;\forall u\in \mathrm{PU},\;\forall \left(\mathrm{ut}\in \mathrm{HUT}\;\wedge \;{\mathrm{UTout}}_{\mathrm{ut}}\geq {\mathrm{Tpinch}}_{\mathrm{ut}}\right)$$

(15) $${\mathrm{UF}}_{\mathrm{pu}}w_{\mathrm{pu},\mathrm{pe}}=\sum_{\mathrm{ut}}{\mathrm{Qu}}_{\mathrm{pu},\mathrm{ut},\mathrm{pe}}+{\mathrm{Qout}}_{\mathrm{pu},\mathrm{pe}}\;\;\;\;\;\;\;\;\;\forall u\in \mathrm{PU},\;\forall \left(\mathrm{ut}\in \mathrm{CUT}\;\wedge \;{\mathrm{UTout}}_{\mathrm{ut}}\leq {\mathrm{Tpinch}}_{\mathrm{ut}}\right)$$

(16) $${\mathrm{Qu}}_{\mathrm{pu},\mathrm{ut},\mathrm{pe}}\leq \sum_{s|\;{\mathrm{Ts}}_{s}\geq \;{\mathrm{Tpinch}}_{\mathrm{pu}}\;\wedge \;{\mathrm{Te}}_{s}\leq {\mathrm{UTout}}_{\mathrm{ut}}}\left({\mathrm{Qh}}_{\mathrm{pu},s}\;-\;{\mathrm{Qc}}_{\mathrm{pu},s}\right)w_{\mathrm{pu},\mathrm{pe}}\;\;\;\;\;\;\forall \mathrm{puPU},\;\forall \;\mathrm{ut}\;\in \mathrm{HUT}$$

(17) $${\mathrm{Qh}}_{\mathrm{pu},s}=\sum_{n|\;{\mathrm{Tin}}_{\mathrm{pu},n}\geq \;{\mathrm{Te}}_{s}\;\wedge \;{\mathrm{Tout}}_{\mathrm{pu},n}\leq {\mathrm{Ts}}_{s}}{\mathrm{MCp}}_{\mathrm{pu},n}\left({\mathrm{Te}}_{s}\;-\;{\mathrm{Ts}}_{s}\right)\;\;\;\;\;\;\;\;\;\forall \mathrm{pu}\in \mathrm{PU};\;\forall s;\forall \mathrm{pe}\in \mathrm{PE}$$

(18) $${\mathrm{Qc}}_{\mathrm{pu},s}=\sum_{n|\;{\mathrm{Te}}_{s}\leq \;{\mathrm{Tout}}_{\mathrm{pu},n}\;\wedge \;{\mathrm{Ts}}_{s}\geq {\mathrm{Tin}}_{\mathrm{pu},n}}{\mathrm{MCp}}_{\mathrm{pu},n}\left({\mathrm{Te}}_{s}\;-\;{\mathrm{Ts}}_{s}\right)\;\;\;\;\;\;\;\forall \mathrm{pu}\in \mathrm{PU};\;\forall s;\forall \mathrm{pe}\in \mathrm{PE}$$

Restrictions (19) and (20) calculate the mass of the ut utility that needs to be supplied, based on the heat demanded from each utility.

(19) $${\mathrm{Qu}}_{\mathrm{pu},\mathrm{ut},\mathrm{pe}}={\mathrm{massUtility}}_{\mathrm{pu},\mathrm{ut},\mathrm{pe}}{\mathrm{hv}}_{\mathrm{ut}}\;\;\;\;\;\;\;\forall u\in \mathrm{PU},\;\forall \mathrm{ut}\in \mathrm{HUT},\;\forall \mathrm{pe}\in \mathrm{PE}$$

(20) $${\mathrm{Qu}}_{\mathrm{pu},\mathrm{ut},\mathrm{pe}}={\mathrm{massUtility}}_{\mathrm{pu},\mathrm{ut},\mathrm{pe}}{\mathrm{hs}}_{\mathrm{ut}}\;\;\;\;\;\;\;\forall u\in \mathrm{PU},\;\forall \mathrm{ut}\in \mathrm{CUT},\forall \mathrm{pe}\in \mathrm{PE}$$

The heat that a process receives from other processes and gives away to other processes is determined by the restrictions (21) to (26) and considers the heat leaving a process pu’ at a stage s (Qs_pu’,s_) and the heat entering a stage s of the thermal cascade of the process pu’ (Qf_pu’,s_)

(21) $${\mathrm{Qout}}_{\mathrm{pu},\mathrm{pe}}=\sum_{s|\;{\mathrm{Te}}_{s}\leq \;{\mathrm{Tpinch}}_{\mathrm{pu}}}{\mathrm{Qs}}_{\mathrm{pu},s,\mathrm{pe}}\;\;\;\;\;\;\;\;\forall \mathrm{pu}\in \mathrm{PU};\;\forall s;\forall \mathrm{pe}\in \mathrm{PE}$$

(22) $${\mathrm{Qin}}_{\mathrm{pu},\mathrm{pe}}=\sum_{s|\;{\mathrm{Ts}}_{s}\geq \;{\mathrm{Tpinch}}_{\mathrm{pu}}}{\mathrm{Qf}}_{\mathrm{pu},s,\mathrm{pe}}\;\;\;\;\;\;\;\;\forall \mathrm{pu}\in \mathrm{PU};\;\forall s;\forall \mathrm{pe}\in \mathrm{PE}$$

(23) $${\mathrm{Qout}}_{\mathrm{pu},\mathrm{pe}}\leq \;\sum_{pu^{‘}|\;{\mathrm{Tpinch}}_{{\mathrm{pu}}^{‘}}>\;{\mathrm{Tpinch}}_{\mathrm{pu}}\;}\sum_{S|\;{\mathrm{Te}}_{s}\leq {\mathrm{Tpinch}}_{\mathrm{pu}}\&{\mathrm{Ts}}_{s}\geq {\mathrm{Tpinch}}_{{\mathrm{pu}}^{‘}}}{\mathrm{Qf}}_{{\mathrm{pu}}^{\prime },s,\mathrm{pe}}\;\;\;\;\;\;\;\;\forall \mathrm{pu}\in \mathrm{PU};\;\forall s;\forall \mathrm{pe}\in \mathrm{PE}$$

(24) $${\mathrm{Qin}}_{\mathrm{pu},\mathrm{pe}}\leq \;\sum_{{\mathrm{pu}}^{‘}|\;{\mathrm{Tpinch}}_{pu^{‘}}<\;{\mathrm{Tpinch}}_{\mathrm{pu}}\;}\sum_{{\mathrm{Te}}_{s}\leq {\mathrm{Tpinch}}_{{\mathrm{pu}}^{\prime }}\&{\mathrm{Ts}}_{s}\geq {\mathrm{Tpinch}}_{\mathrm{pu}}}{\mathrm{Qs}}_{{\mathrm{pu}}^{\prime },s,\mathrm{pe}}$$

(25) $${\mathrm{Qf}}_{\mathrm{pu},s,\mathrm{pe}}\left\{\begin{array}{c} =0\\ \leq \left({\mathrm{Qc}}_{\mathrm{pu},s}-\;{\mathrm{Qh}}_{\mathrm{pu},s}\right)w_{\mathrm{pu},\mathrm{pe}} \end{array}\right.$$

(26) $${\mathrm{Qs}}_{\mathrm{pu},s,\mathrm{pe}}\left\{\begin{array}{c} =0\\ \leq \left({\mathrm{Qh}}_{\mathrm{pu},s}-\;{\mathrm{Qc}}_{\mathrm{pu},s}\right)w_{\mathrm{pu},\mathrm{pe}} \end{array}\right.$$

The heat entering stage s of the thermal cascade of the pu process is limited by the heat demanded by that stage, as shown in Equation (25). Similarly, Equation (26) guarantees that the heat leaving stage s of the thermal cascade of the pu process is limited by the excess heat from stage s. In order to ensure compliance with the rules of the Pinch methodology, stages located above the pinch have a Qs value equal to zero, while stages located below the pinch temperature also have their Qf values set to zero. The Equation (27) guarantees that the total heat input into the processes is equal to the total heat that left the processes in that period pe, respecting the consideration that heat is not a resource that can be stored.

(27) $$\sum_{\mathrm{pu}}{\mathrm{Qin}}_{\mathrm{pu},\mathrm{pe}}=\sum_{\mathrm{pu}}{\mathrm{Qout}}_{\mathrm{pu},\mathrm{pe}}$$

#### 2.1.7. Process Capital Cost and Investment Cost Linearization

As previously mentioned, each process is inserted into the superstructure as a reference capacity and in the form of a surrogate model. Although resource consumption and production vary linearly with the scale of the process, the investment cost tends to vary non-linearly in relation to its capacity. To maintain the linearity of the model, a piecewise linearization of the cost curve for each process was carried out. To achieve this, each cost curve was divided into different levels, each limited by a maximum (capMax_u,l_) and minimum (capMin_u,l_) value.

When a process is selected to make up the biorefinery, only one of the levels is chosen. It was therefore necessary to rewrite Equation (1) in the form of Equation (28) and introduce the auxiliary constraints (29) to (31). The lower limit at level l is represented by ${CapMin}_{u,l}$, while ${CapMax}_{u,l}$ represents the upper limit that the scale adjustment variable can have in interval l. The binary variable ${ylevel}_{u,l}$ takes on a value of one when that level is selected or zero when it is not selected. When selected, ${ylevel}_{u,l}$ has a value of one and the variable ${wlevel}_{u,l}$ has a value between the maximum and minimum of that level. When not selected, both ${ylevel}_{u,l}$ and ${wlevel}_{u,l}$ for that level have a value of zero.

Constraint (30) guarantees that only one level can be selected when a unit is chosen to make up the biorefinery. Thus, when a unit is selected, the scale adjustment variable ($w_{u,pe}$) will display the value of the do ${wlevel}_{u,l}$ of the selected level. The investment cost is given by Equation (32).

(28) $${CapMin}_{u,l}{yLevel}_{u,l}\;<\;{wlevel}_{u,l}\;\leq \;{CapMax}_{u,l}{yLevel}_{u,l}$$

(29) $${yLevel}_{u,l}\leq y_{u}$$

(30) $$\sum_{l}{yLevel}_{u,l}=y_{u}$$

(31) $$w_{u,pe}-\sum_{l}{wlevel}_{u,l}\leq 0$$

(32) $${invCost}_{u}=\sum_{l}{capCostA}_{u,l}{wlevel}_{u,l}+{capCostB}_{u,l}{ylevel}_{u,l}$$

#### 2.1.8. Objective Function

The proposed optimization problem has two evaluation criteria, environmental and financial, that can be used alone or together to generate the pareto curve. As an economic criterion, the formulation considers the net present value (NPV) maximization (OF1), Equation (33), determined by Equations (34)–(41). Investment is the total investment and takes into account the investment in process and storage for resources (storageCost_r_), which is determined based on the unit investment cost (${STCost}_{r}$) and maximum quantity stored (${storageLevel}_{r}$). cashIn and cashOut represent the cash inflow and outflow of location p, respectively. The formulation assumes that the cash flow starts in year one (t = 1) and remains constant until year 20 (t = 20), at a rate of 10% (i = 10%). The cash inflow is considered to be the revenue from the production of the resource, Equation (38), where ${MP}_{r}$ is the selling price of resource r. The cash outflow considers the resources consumed by the processes (RMCost) and other costs (OtherCost), the value of which is set at 10% of the total investment in location p.

(33) maxOF1

(34) $$OF1=\sum_{p}{VPL}_{p}$$

(35) $$VPL=-investment+\sum_{t}\frac{cashIn-cashOut}{{\left(1+i\right)}^{t}}$$

(36) $$investment=\sum_{u}{invCost}_{u}+\sum_{r}{storageCost}_{r}$$

(37) $${storageCost}_{r}={storageLevel}_{r}{STCost}_{r}$$

(38) $$cashIn=\sum_{u}\sum_{r}\sum_{pe}{prod}_{u,r,pe}{MP}_{r}$$

(39) cashOut=RMCost+OtherCost

(40) $$RMCost=\sum_{u}\sum_{r}\sum_{pe}{cons}_{u,r,pe}{RMP}_{r}$$

(41) $${storageLevel}_{r}\geq {stockNetF}_{r,pe}$$

To consider the environmental aspect, the maximization of total CO_2_ avoided was considered, Equation (42), which takes into account the emissions from the purchase and transport of the resource and the amount avoided by replacing the fossil fuel with its equivalent biofuel (${av{CO}_{2}}_{r}$). The emissions from the purchase consider the quantity purchased and the specific emissions of that resource (${{emCO}_{2}}_{r}$).

(42) maxOF2

(43) $$OF2={CO}_{2}avoided-{emittedCO}_{2}$$

(44) $${CO}_{2}avoided=\sum_{pe}\sum_{r}{sold}_{r,pe}{av{CO}_{2}}_{r}$$

(45) $${emitted\;CO}_{2}=\sum_{pe}\sum_{r}{bought}_{r,pe}{{emCO}_{2}}_{r}$$

#### 2.1.9. Generating the Pareto Curve

To generate the Pareto curve, a computational procedure was implemented that first determines the ends of the curve and then connects them using Equation (47), which is based on the ε-constraint method [11]. The main objective function was to maximize OF1. max_OF2 and min_OF2 are the maximum and minimum values of OF2, obtained at the environmental optimum and economic optimum, respectively, while nCase is the number of points connecting the ends of the Pareto curve.

(46) $$\varepsilon =\frac{\mathrm{max\_OF}2-\mathrm{min\_}OF2}{nCase-1}$$

(47) OF2=OF2a+ε

**Figure 3:**
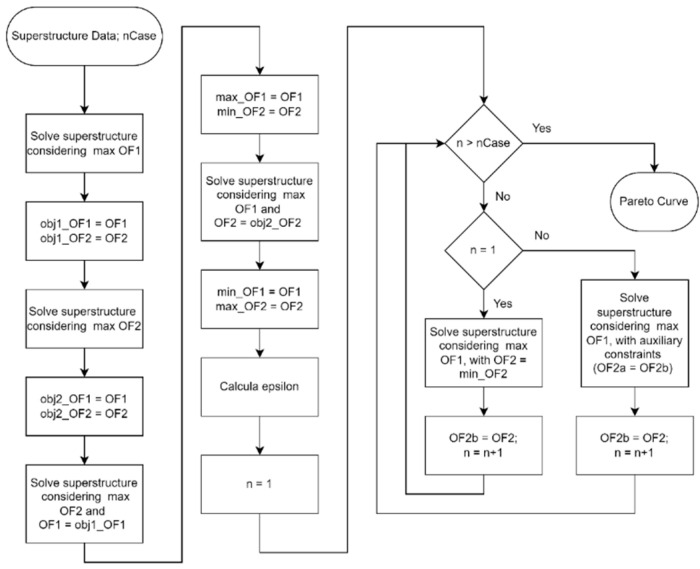
Representation of the computational procedure for obtaining the Pareto curve.

**Figure 4:**
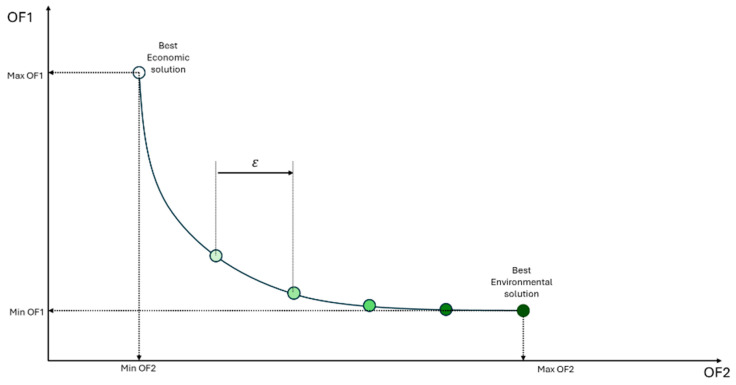
Representation of a Pareto curve generated using the described procedure.

Figure 3 is a representation of the computational procedure used to generate the Pareto curve. Initially, the superstructure is solved considering objective function 1, which refers to the economic aspect, as the main function, and the values of the solution’s performance variables, OF1 and OF2, are stored. Next, the superstructure is solved again, this time considering objective function 2, related to the environmental aspect, as the main function, and the values of the performance variables are again stored. These two resolutions represent the economic and environmental optimum points, respectively. However, it is necessary to ensure that, for the environmental optimum, the value of OF1 is the maximum for that value of OF2, and that, for the economic optimum, the value of OF2 is really the maximum for that value of OF1. To guarantee this, the superstructure is solved again, but by fixing the value of OF1 at obj1_OF1 and considering objective function 2 as the main one. By maximizing OF2 with OF1 fixed at its maximum value, it can be said that the solution obtained is the economic optimum point [11]. Next, the superstructure is solved by maximizing the value of OF1 while OF2 is fixed at obj2_OF2. By maximizing OF1 with OF2 fixed at its maximum value, it can be said that the solution obtained is the environmentally optimal point. With the two ends of the Pareto curve determined, the economic optimum and the environmental optimum, it is possible to determine the value of ε, which represents the spacing between the points that will be generated and connect the two ends of the curve. Then, starting from the economic optimum, the superstructure is solved several times, always with the value of OF2 fixed based on the previous resolution and incremented by epsilon. When solving the superstructure maximizing OF1 and considering increasing OF2 values, the value of OF1 approaches its value at the environmental optimum point until it equals it. At this point, when the environmental optimum point is reproduced, the two ends of the Pareto curve are connected, and the curve is finalized. Figure 4 shows a generic representation of the Pareto curve obtained using the procedure presented.

#### 2.1.10. Key Performance Indicator

To further compare the configurations of the different cases, performance indicators were established for net energy production (NEP), energy efficiency (η), and net energy production per area (NEPA), calculated using Equations (48)–(50), respectively. LHV_r_ represents the lower heating value for the biofuels, total sugarcane area, and total photovoltaic solar panel area, representing the total area necessary to produce the consumed sugarcane and electricity. To calculate the total sugarcane area, the productivity value of 80 tons of sugarcane per hectare was used, while for the total photovoltaic solar panel area, the value of 5 kWh/m^2^.day with an efficiency of 20% was considered.

(48) $$NEP=\sum_{r}\sum_{pe}{sold}_{r,pe}.{LHV}_{r}-\sum_{r}\sum_{pe}{bought}_{r,pe}.{LHV}_{r}$$

(49) $$\eta =\frac{\sum_{r}\sum_{pe}{sold}_{r,pe}.{LHV}_{r}}{\sum_{r}\sum_{pe}{bought}_{r,pe}.{LHV}_{r}}$$

(50) $$NEPA=\frac{\sum_{r}\sum_{pe}{sold}_{r,pe}.{LHV}_{r}-\sum_{r}\sum_{pe}{bought}_{r,pe}.{LHV}_{r}}{Total\;Sugarcane\;area+Total\;solar\;painel\;area}$$

## 3. Case Study: Integrating Biomethanol Production into Sugarcane Biorefinery

As mentioned above, this study aims to evaluate the best strategies for integrating methanol production into a sugarcane biorefinery. The main objective is to identify the configurations that simultaneously optimize the system’s economic and environmental performance, as well as to evaluate the impact of energy integration between processes. To this end, we used a superstructure based on Mixed Integer Linear Programming (MILP), capable of considering mass and energy balances, energy integration, and process selection and sizing in an integrated manner. In addition, to verify the impact of heat recovery between processes on the configuration of the biorefinery, two scenarios were evaluated: with and without energy integration between processes. The model uses the maximization of the net present value (NPV) and the maximization of the total amount of CO_2_ avoided as objective functions.

In both cases evaluated in this article, a conventional autonomous sugarcane distillery with a typical milling capacity of 2,640,000 tons of sugarcane per year (500 tons per hour) was considered, producing ethanol; bagasse; vinasse; filter cake; and CO_2_. Biomethanol can be produced via gasification of bagasse or catalytic hydrogenation of CO_2_. In addition, other technologies and auxiliary processes have been considered to produce the resources needed to operate the biorefinery. For hydrogen production, the processes of biomethane steam reforming, ethanol steam reforming, and alkaline water electrolysis were evaluated. Biomethane can be produced via biodigestion of vinasse and/or filter cake. For steam generation, three simple Rankine cycles with steam condensation at 9, 6 and 2 bar pressure at the turbine outlet and two boilers were considered, with each Rankine cycle representing a different cogeneration system. Electricity can be supplied by the electricity grid, a photovoltaic plant, or a power plant, in addition to cogeneration systems.

Table 1 shows the main characteristics of the processes and the resources consumed and produced. To ensure that the biorefinery’s economic and environmental performance is not limited, sufficiently high demand values were used for the resources: ethanol, electricity, methane, methanol and CO_2_. In this way, the biorefinery would be free to opt for the most economically and/or environmentally advantageous production. About availability, a value high enough to ensure that resources such as photovoltaic electricity and water are not depleted was adopted. For the availability of sugarcane, a value of 87,828,026 tons was adopted, equivalent to the production of this raw material in the São José do Rio Preto mesoregion, in the state of São Paulo, in the year 2023 [22]. Tables S1–S4 show, respectively, the linearized cost curves, the thermal currents considered, the purchase and sale prices of the resources, and the emissions associated with the purchase and sale.

In this work, the selection of candidate processes for the superstructure was guided by technical feasibility within the current industrial context of a sugarcane biorefinery. The hydrogen production routes considered were chosen to take advantage of resources that are already available at the mill, thus ensuring technological coherence with the existing infrastructure. In addition, bagasse gasification was included because it is widely recognized as one of the most promising novel routes for biomethanol production, with high potential to unlock additional value creation from biomass in future biorefineries [23].

**Table 1:** Main characteristics of the processes.

Process	Main Characteristics	Input Resources	Output Resources	Data Sources
Sugarcane Distillery (SD)	- Milling capacity: 500 ton/h - Cleaning; preparation; extraction system; treatment and concentration; sterilization and fermentation; distillation and rectification - Dehydration step with MEG	- Sugarcane - Water - Electricity - Heat	- Ethanol Anhydrous - Vinasse - Filter Cake - CO_2_ - Bagasse	[24,25]
Cogeneration systems (LP; MP; and HP)	- Steam-based cycle with steam turbines - Cogeneration efficiency: 85% - Bagasse net calorific value: 7500 kJ.kg^−1^	- Sugarcane bagasse	- Steam (LP: 2.2 bar; MP: 6 bar; and HP: 9 bar) - Electricity	[18]
Bagasse powerplant (BP)	- System efficiency: 35% - Bagasse net calorific value: 7500 kJ.kg^−1^	- Sugarcane bagasse	- Electricity	[18]
Vinasse biodigestion (VBD)	- Single-phase biodigestion - OLR: 25 kgCOD.m^−3^.day^−1^ - COD removal: 60.7%; - Methane Production: 0.234 Nm^3^. kg^−1^COD_removed_	- Vinasse - Electricity	- Biomethane - CO_2_	[26]
Filter cake biodigestion (FCBD)	- Single-phase biodigestion - Total solids concentration: 30% - Volatile solids concentration: 75% - Methane yield: 0.26 Nm^3^.kg^−1^ VS - Methane purification by PSA	- Filter cake - Electricity	- Biomethane - CO_2_	[26,27]
Bagasse gasification (BG)	- Indirect gasification - Main process steps: bagasse pretreatment and gasification; syngas conditioning; methanol synthesis, and upgrading - Residual gases from gasification;	- Sugarcane bagasse	- Methanol	[28,29]
Catalytic CO_2_ Hydrogenation (CCH)	- Main process steps: CO_2_ and H_2_ compression; methanol synthesis; methanol upgrade	- CO_2_ - H_2_ - Electricity	- Methanol	[30,31]
Electrolysis (E-PEM)	- Proton exchange membrane technology	- H_2_O - Electrolysis	- H_2_;	[32]
Steam Methane Reforming (SMR)	- Main process steps: methane purification; reforming; syngas adjustment; hydrogen purification (PSA technology) - Methane and residual gas stream as fuel	- CH_4_ - Electricity	- H_2_;	[33]
Steam Ethanol Reforming (SER)	- 20-atm conventional tube-in-shell SR with PSA gas cleanup - Ethanol is used as supplemental fuel for the burner	- Ethanol - Electricity - Water	- H_2_;	[34]
Bagasse Boiler	- Bagasse net calorific value: 7500 kJ.kg^−1^	- Sugarcane Bagasse	- Steam (LP: 2.2 bar; and MP: 6 bar);	- Modeled by the authors

LR—Organic Loading Rate; COD—Chemical Oxygen Demand; PSA—Pressure Swing Absorption; VS—Volatile Solids; SR—Steam Reactor; LP—Low Pressure; MP—Medium Pressure; HP—High Pressure.

## 4. Results and Discussion

**Figure 5:**
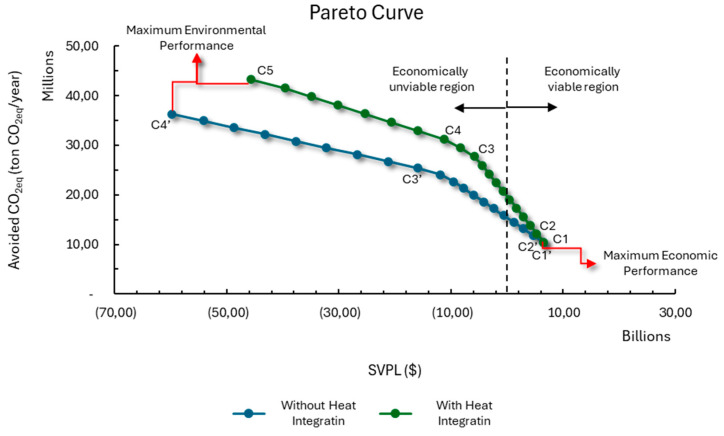
Representation of the Pareto curves obtained for the case with and without energy integration between processes, where the points C1–C5 is the configuration with heat integration, and the points C1’–C4’ is the configuration without heat integration.

In this initial stage of the study, the focus was on a detailed analysis of the various possible configurations for the biorefinery, seeking to understand how it can achieve the best environmental performance from its economic optimum point, as well as the effects provided by heat integration between processes, especially in relation to the use of recovered heat. To this end, it was assumed that both the resources available and those required would be concentrated in the same location, thus eliminating logistical influences that could interfere with the results. Two scenarios were then evaluated—with and without heat integration. The Pareto curves illustrating the results obtained in these two contexts are shown in Figure 5. Looking at the values on the curve, it is possible to see that the objectives being assessed are antagonistic, meaning that improving the biorefinery’s environmental performance implies penalizing its economic performance, which could even result in the biorefinery becoming economically unviable. With this, it is possible to separate the solutions that make up the Pareto curve into two sets, one made up of economically viable solutions, with an NPV greater than zero, and the other of unviable solutions. When we compare the curve for the case with heat integration and the case without heat integration, it is possible to see that the case with heat integration will always have higher environmental performance than the case without heat integration. This improvement in environmental performance can be explained by the fact that by making it possible to recover heat between processes, it is possible to use bagasse as a raw material for direct fuel production, and not just as fuel, as will be further explained throughout this work.

The impact of heat integration on the environmental and economic performance of the biorefinery becomes more evident when drawing a horizontal line connecting the environmental extreme of the case without heat integration to the Pareto curve of the case with heat integration, as shown in Figure S1. This shows that energy integration makes it possible to achieve the same environmental performance at a lower economic cost. Without heat recovery between processes, the biorefinery relies exclusively on boilers or cogeneration systems to meet the heat demand, which requires the use of bagasse as fuel. However, with energy integration, bagasse can be used simultaneously to generate heat and produce methanol, optimizing the use of resources and improving its economic and environmental performance.

Table 2 shows the values obtained for the two objective functions for the extremes of the Pareto curve in the two cases evaluated. The curves coincide at the point of maximum economic performance but diverge as their environmental performance improves. Although heat integration did not generate any financial or environmental gains at the economic optimum, the transfer of heat between processes led to a 19.28% increase in the balance of CO_2_ avoided at the environmental extreme.

By checking the technologies present in the biorefinery along both Pareto curves, it is possible to observe the entry and exit of processes as the biorefinery migrates from one extreme to the other. Figure 5 shows the location of the points where there is a change in the configuration of the biorefinery.

**Figure 6:**
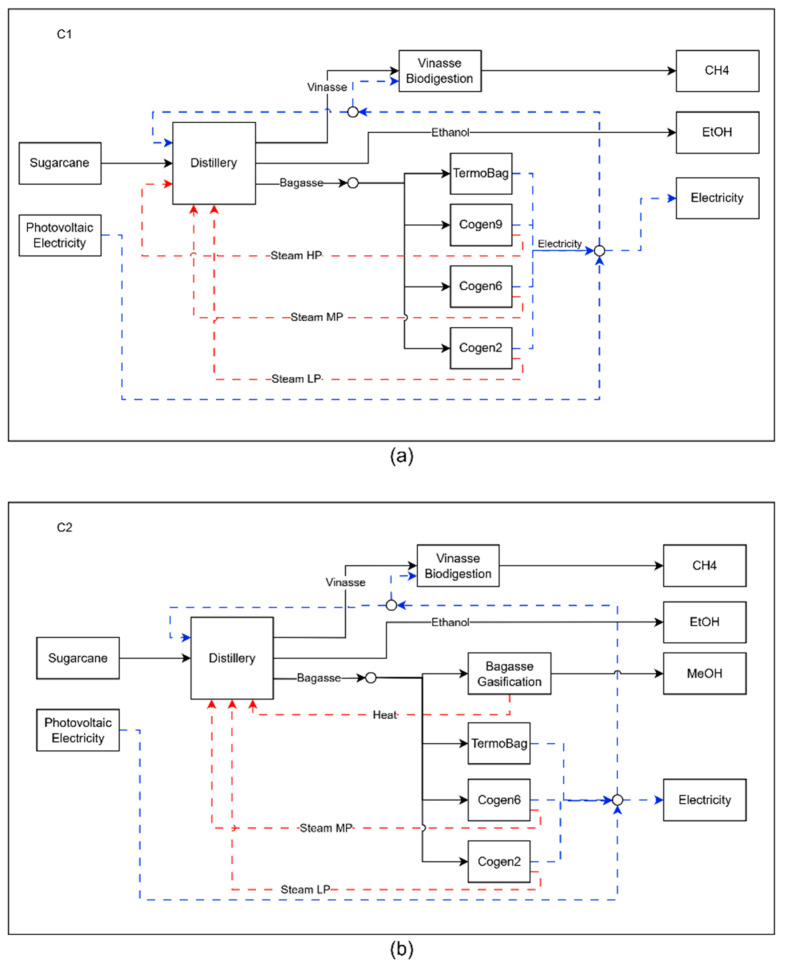
Representation of the biorefinery configuration for cases C1 (**a**) and C2 (**b**), where the dashed blue lines represents electricity and the red lines represent heat flow.

Configuration 1 (C1), shown in Figure 6a represents the most economically attractive technological arrangement for the plant. To achieve maximum economic performance, all the bagasse generated by the biorefinery is directed to the cogeneration systems, which produce all the steam consumed by the process. Since the capacity of the cogeneration systems is limited by the plant’s steam consumption, there is a surplus of bagasse that is sent to the thermoelectric plant. Unlike the other units in the biorefinery, which typically shut down during the off-season, the bagasse powerplant remains operational throughout this period. This continuous operation allows for the utilization of surplus bagasse beyond the crop season, leading to more efficient use of the capital invested and contributing to the overall economic performance of the biorefinery. Except for the presence of the vinasse biodigestion process, the biorefinery has a configuration very close to that found in ethanol plants in Brazil [35]. Although it is not yet common in the sector, vinasse biodigestion has been introduced in mills as a result of technological maturity and the creation of policies that stimulate the production of biofuels, such as the RenovaBio [36,37].

With the prioritization of environmental performance by the biorefinery, in configuration C2, part of the bagasse that was used to generate electricity is now converted into biomethanol through the gasification process. Although methanol has a higher CO_2_ avoided value than electricity, just 7% of bagasse is sent to gasification, since the profitability per ton of bagasse is higher at the thermoelectric plant (33.97 USD/ton bag) compared to gasification (19.55 USD/ton bag). With the heat transfer from the gasification process, the distillery began to consume less steam, leading to the deactivation of the 9-bar cogeneration system, which had the lowest efficiency in generating electricity per ton of bagasse. As in configuration C1, the bagasse powerplant operates fully during the harvest and off-season periods, which also occurs with the gasification process in C2. Although there is a surplus of unused heat during the off-season period and it is necessary to invest in bagasse storage, by building a smaller gasification process that operates continuously throughout the year, it was possible to reduce the total investment by USD 2255.54 × 10^6^.

**Figure 7:**
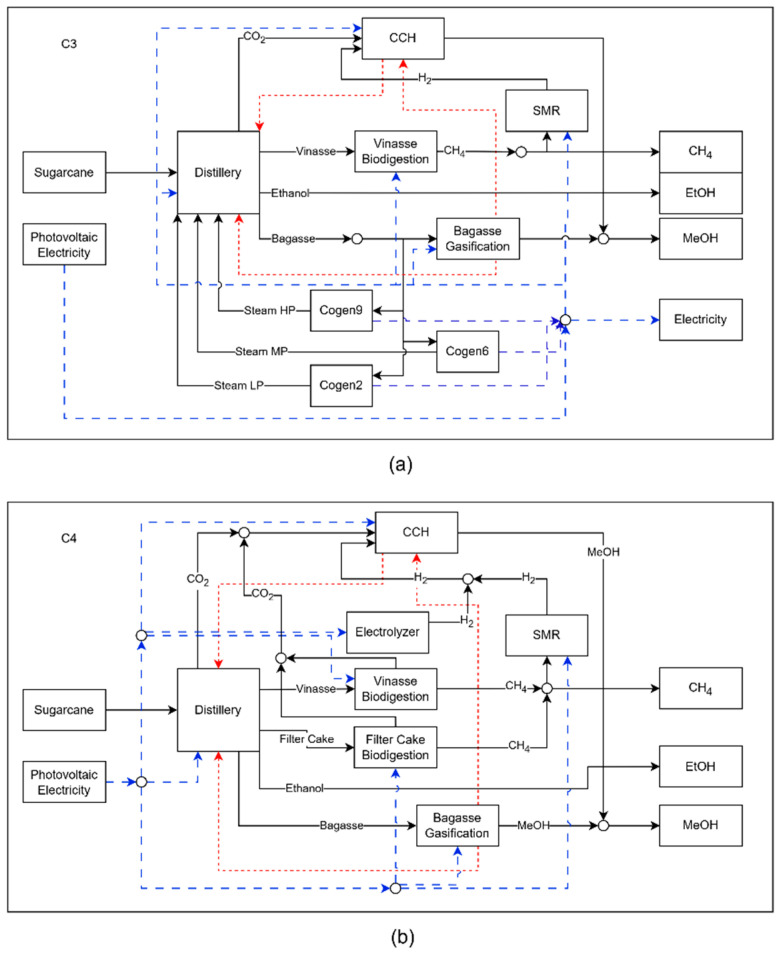
Schematic representation of biorefinery configuration in C3 (**a**) and C4 (**b**), where the dashed blue lines represents electricity and the red lines represent heat flow.

To increase methanol production, the technology for converting CO_2_ into methanol, CCH, enters the biorefinery in the C3 configuration. As this technology consumes hydrogen (H_2_), of the available alternatives, methane reforming proved to be the most suitable, despite consuming practically all of the biomethane produced through the biodigestion of vinasse. Unlike the gasification process, the conversion of CO_2_ into methanol requires heat in its operations, which is supplied by the gasification process, as can be seen in Figure 7a. Although the scale of the gasification process is larger than that of the C2 configuration, this process is still unable to supply all the heat demanded by the biorefinery, making it necessary to activate the cogeneration systems to supply the heat, in the form of steam, that is still demanded by the distillery. Although the distillery consumes all the steam produced, it still receives 1345.09 MW of heat released by the gasification unit and CCH, while CCH receives 35.39 MW from gasification. The gradual increase in the scale of the gasification process, between the C2 and C3 configurations, and only then the entry of new technologies, is an indication that heat recovery between processes and the use of bagasse should be prioritized when the aim is to improve the environmental performance of the biorefinery, with the least economic impact. This is in line with the findings of various studies, such as Jiao et al., who managed to reduce emissions from the extractive distillation and pressure flotation extractive distillation processes by 26% and 30.48%, respectively, after energy integration [38].

Configuration C4, shown in Figure 7b, differs from its predecessor by eliminating the cogeneration systems and incorporating PEM electrolysis and filter cake biodigestion technologies. Unlike vinasse, filter cake can be stored and used during the off-season, enabling continuous biomethane production even when the plant is not in operation. This feature allows the CCH process to operate for longer periods, increasing methanol production due to the availability of CO_2_. The integration of electrolysis leads to a substantial rise in electricity consumption, now fully met by photovoltaic energy. However, in contrast to configurations C2 and C3, the bagasse gasification unit in C4 does not operate continuously throughout the year. With the removal of cogeneration, gasification becomes the primary source of thermal energy for the biorefinery, requiring expanded capacity to meet steam demand during the harvest season. As more bagasse is consumed in this period—without an increase in sugarcane processing or additional bagasse supply—the system faces a shortage of raw materials in the off-season, forcing the shutdown of gasification operations during part of that period. Although configuration C4 enhances overall methanol output, the intermittent use of the gasification process negatively impacts the economic performance of the biorefinery and, as a result, diminishes the competitiveness of the produced biofuels.

**Figure 8:**
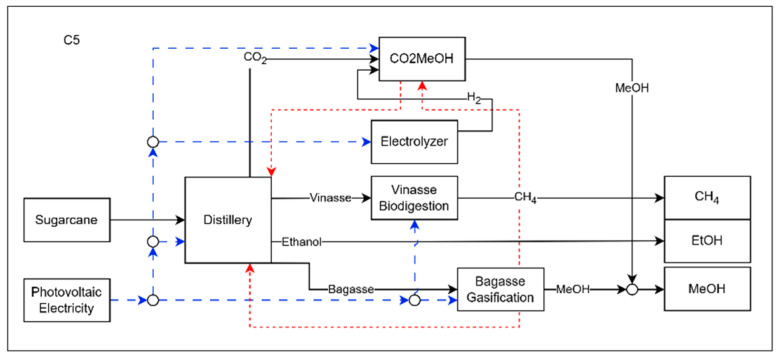
Representation of the C5 configuration of the biorefinery, where the dashed blue lines represents electricity and the red lines represent heat flow.

Configuration C5, shown in Figure 8, represents the biorefinery configuration with the highest environmental performance. In this configuration, all the resources produced are used in such a way as to maximize the production of biofuels. Both vinasse and filter cake are sent to the biodigestion process, generating biomethane and CO_2_. This CO_2_, combined with that released by the plant’s fermentation vats, is converted into methanol via the CCH process, which in turn transfers part of its surplus heat to the distillery. Unlike the previous cases, all the hydrogen consumed is produced by the electrolysis of water, which consumes electricity purchased from a photovoltaic system, just like the other processes in the biorefinery. The bagasse is used entirely for the gasification process, the excess heat from which is transferred to the CCH and the distillery. This integration optimizes heat recovery and the plant’s environmental performance, consolidating the C5 configuration as the most sustainable model.

Tables S5 and S6 show the list of the processes that make up the biorefinery for the cases with and without heat integration in each configuration evaluated. Unlike the configurations with energy integration, in all the configurations without energy integration, part of the bagasse is used as fuel to supply heat, either through a cogeneration system or a boiler, as in configurations C3’ and C4’.

**Figure 9:**
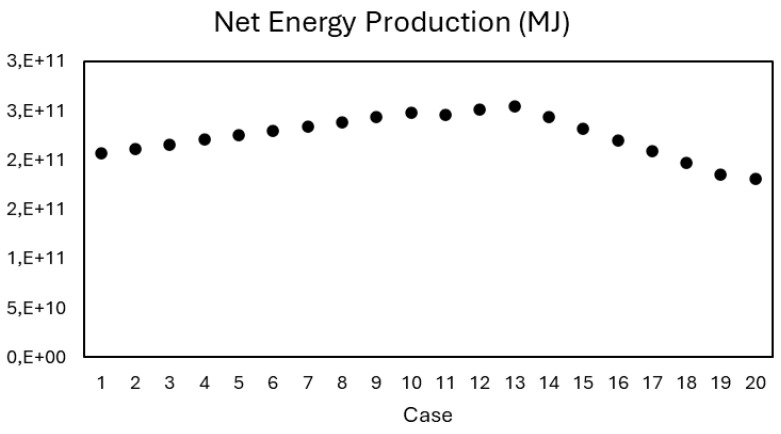
Net Energy Production curve for the case with heat integration.

Figure 9 presents the net energy production (NEP) curve for the scenario with heat integration. A continuous increase is observed up to C4, followed by a gradual decline through C5. The initial growth can be attributed to the reallocation of bagasse from the thermoelectric plant—whose energy efficiency is approximately 35%—to the gasification process, which offers a significantly higher efficiency of around 65%. This technological shift enables more effective use of the energy content in bagasse, enhancing biofuel production and improving the overall energy performance of the biorefinery.

Initially, the biorefinery exhibits an energy efficiency of 46.3%, which gradually increases to a maximum of 57.1%. This trend coincides with the scale-up of the gasification process, reinforcing the relationship between technology choice and energy performance improvement. Following configuration 13, both NEP and energy efficiency begin to decline. From this point onward, hydrogen production is introduced through water electrolysis, which increases electricity demand by 91%. Although electrolyzers operate at an energy efficiency of approximately 64%, when combined with the efficiency of the downstream CCH process, the overall system efficiency drops to 48.13%, leading to a decrease in the biorefinery’s total energy efficiency. Despite this, the net energy production per area (NEPA) continues to rise throughout all configurations. This is because, even with the increased electricity demand, the land area required for solar panels is significantly smaller than the area of sugarcane needed to produce the same amount of energy. While solar panels yield about 1314 MJ/m^2^·year, sugarcane delivers only 40.69 MJ/m^2^·year—demonstrating more than thirty times the energy productivity per unit of land.

**Figure 10:**
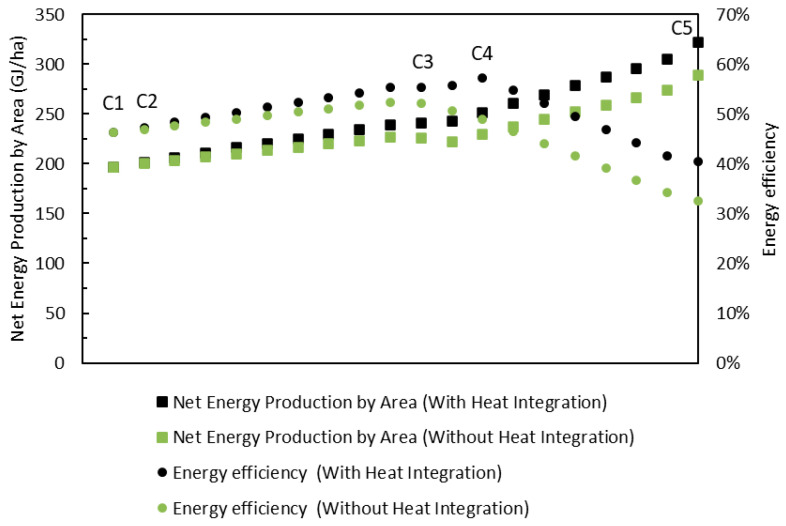
NEPA and efficiency curves for the cases with and without heat integration.

The analysis of the curves for the case without thermal integration, shown in Figure 10, shows that the biorefinery reaches a maximum efficiency of 52.4%, which is 5.8% lower than the scenario with integration. There is also a general drop in the performance of the configurations evaluated. Initially, both scenarios show the same results, as they share the same configuration. However, as the solutions progress towards the configurations with the greatest environmental benefit, the curves begin to move apart—precisely at the point where less bagasse is sent to cogeneration in the case with thermal integration. The same pattern is observed in net energy production per area, with a 10.65% reduction in the maximum value obtained when there is no thermal integration. As highlighted above, without heat recovery, the biorefinery needs to keep part of the bagasse in the cogeneration to meet steam demand, which limits redirection to gasification and, consequently, reduces biomethanol production and the energy use of bagasse. These results reinforce the importance of maximizing the recovery of excess heat in order to increase the efficiency and competitiveness of the biorefinery. Table 3 shows the maximum values obtained for the performance indicators.

### 4.1. Change in NPV as a Function of the Change in the Total Amount of CO_2_ Avoided

**Figure 11:**
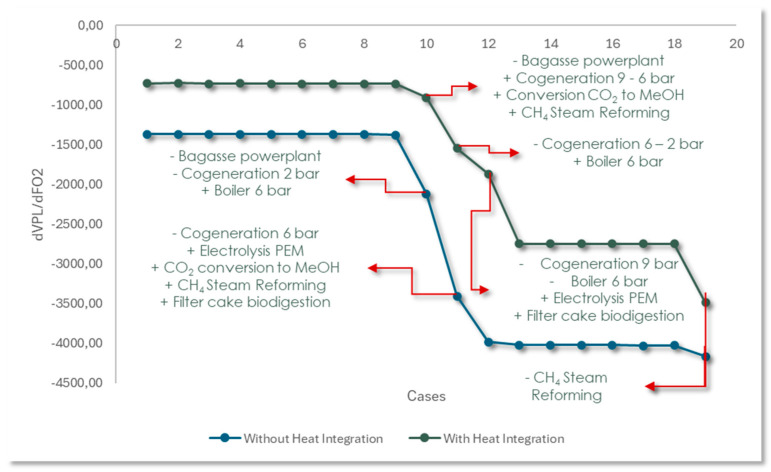
NPV variation curves as a function of avoided CO_2_ variation.

When analyzing the variation curve of the net present value (NPV) in relation to the amount of CO_2_ avoided, shown in Figure 11, the slope varies throughout the different regions. In some regions, the slope is subtle, while in others, it is steeper. These changes reflect crucial moments in the Pareto curve. Regions with little variation indicate scale adjustments or the decommissioning of technologies with limited impact, such as cogeneration systems. On the other hand, pronounced variations correspond to the inclusion or exclusion of technologies with a greater impact, such as the electrolyzer, which demands a large amount of electricity without directly generating biofuels. On the curve with heat integration, points 10, 11, and 12 highlight the transition to more efficient processes, such as the replacement of bagasse power plants with technologies for converting CO_2_ into methanol, CH_4_ reforming, filter cake biodigestion, and PEM electrolysis. In the curve without heat integration, similar changes occur at points 10 and 11. However, due to the smaller range of cases analyzed, these changes result in a steeper slope at these specific points.

### 4.2. Avoided CO_2_ Cost

**Figure 12:**
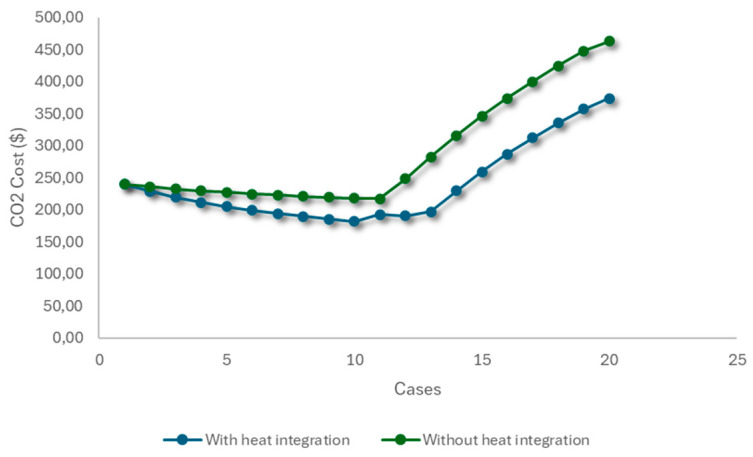
Evolution of the cost of CO_2_ avoided over the cases generated.

Figure 12 shows the cost of each ton of CO_2_ avoided for the points that make up the Pareto curve. There is a significant reduction between cases 1 and 10 when there is energy integration. Despite the increase in investment, operating and raw material costs, as shown in Figure S2, the rise in raw material costs is less pronounced in relation to the amount of CO_2_ avoided, resulting in a lower cost per ton. However, between points 10 and 20, production costs increase due to the underutilization of gasification and the introduction of the electrolyzer in the biorefinery. Without energy integration, costs are higher because a portion of the bagasse needs to be used as fuel for utility generation, reducing methanol production and, consequently, the amount of CO_2_ avoided.

### 4.3. Revenue Composition

**Figure 13:**
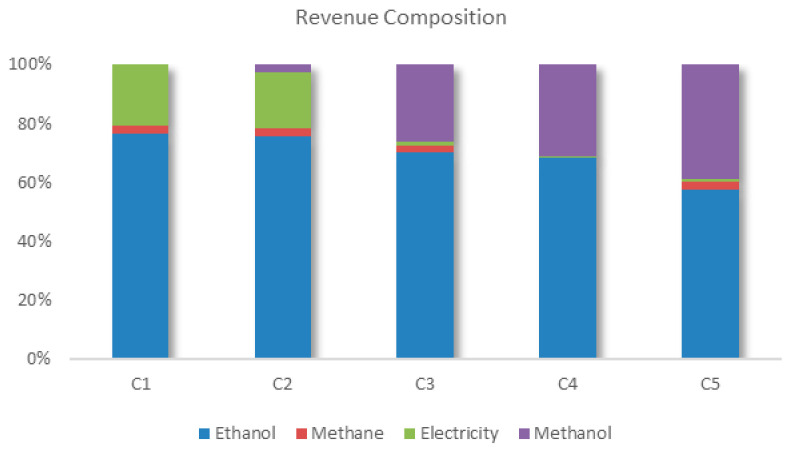
Composition of revenue for the different biorefinery configurations found.

In the maximum economic performance scenario, the main revenue comes from the sale of ethanol and electricity, with a smaller contribution from methane, as can be seen in Figure 13. These products are the same as those marketed in most of the sector’s current units. Within the context evaluated, the sale of ethanol remains constant throughout the configurations. However, as environmental performance is prioritized, the contribution of methanol becomes more significant, while that of electricity decreases. This change can be justified by the external supply of electricity to the biorefinery, and the higher CO_2_ avoided value of methanol.

In this configuration, the biorefinery buys the amount of electricity it needs for its processes from an external photovoltaic system and sells the electricity generated by the cogeneration and powerplant systems. This scenario suggests that, under the conditions evaluated, it may be more advantageous to purchase photovoltaic electricity to meet internal demands and sell the electricity produced internally. This relationship between consumption and production can be justified by considerations of purchase and sale prices and CO_2_ emissions. As both sources of electricity have the same purchase price, the cost of electricity for the processes is indifferent to its origin. Furthermore, by purchasing photovoltaic electricity, the system has the possibility of commercializing all the electricity generated, thus increasing the amount of CO_2_ avoided by the biorefinery. Furthermore, methanol has a higher CO_2_ avoided value than electricity, making the use of bagasse to produce methanol more environmentally advantageous in the competitive context of this resource. So, the importance of methanol grows as environmental performance is prioritized, making it the main component of revenue.

An analysis of the commercialization of biomethane reveals a very low share of revenue in configuration 1, which is maintained in configuration 2. In configuration 3, it is reduced due to the consumption of biomethane to produce the hydrogen needed for the process of converting CO_2_ into methanol. Four technologies were considered to meet the hydrogen demand: biomethane reforming, ethanol reforming, a PEM electrolyzer, and an alkaline electrolyzer. Initially, biomethane reforming proved to be the most advantageous alternative, redirecting CH_4_, which would have been sold, to hydrogen production. However, as the conversion of CO_2_ into methanol increases, other sources of hydrogen must be integrated into the biorefinery to compensate for the shortage of biomethane. Although the biorefinery acquires electricity from a photovoltaic plant, electrolysis technologies have high electricity consumption, impacting the system financially and delaying the incorporation of this technology into the biorefinery. Another notable aspect is the absence of ethanol reforming technology from the Pareto curve at any time. It is important to note that, like bagasse, there is competition for the use of ethanol, which can be sold or used to produce hydrogen. When destined for reforming, the displacement of ethanol use must be considered. As well as reducing the biorefinery’s revenue due to the lack of commercialization, the detour of ethanol to hydrogen production also penalizes the total amount of CO_2_ avoided by the biorefinery. For every ton of hydrogen produced by reforming ethanol, the biorefinery stops marketing 5.6 tons of ethanol, resulting in a reduction of USD 3775.90 in revenue and 14.78 tons of CO_2_ not avoided.

Different authors and institutions point to the lack of competitiveness of biofuels as one of the main causes for the unfeasibility of biorefineries. As previously mentioned, when evaluating the economic performance of the biorefinery, it can be seen that most of the configurations on the Pareto curve are located in a region of economic unfeasibility, i.e., they have an NPV less than or equal to zero, as shown in Figure 5. This suggests that improving the environmental performance of the biorefinery could have a negative impact on the viability of the system, which could lead to production becoming unfeasible. One strategy that can be used to compensate for the economic impact of increased environmental performance would be to remunerate the production of biofuel, based on its sale and consumption, based on the amount of CO_2_ equivalent that is no longer emitted. To compensate for this penalty, in the two cases evaluated and along the Pareto curve, remuneration was added to the biorefinery’s revenues based on the amount of CO_2_ equivalent that was avoided. The price that should be paid for each ton of CO_2_ avoided was determined in two contexts: (I) bringing the NPV to zero, and (II) maintaining maximum economic performance.

**Figure 14:**
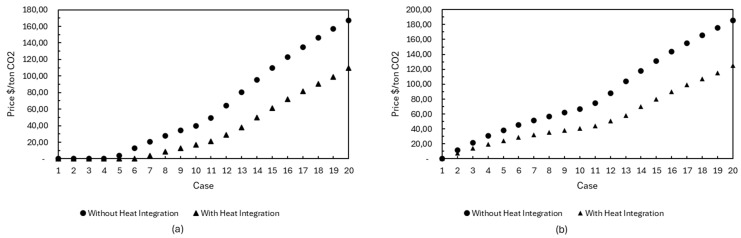
Price to be paid for each ton of CO_2_ equivalent that is no longer emitted for the cases evaluated, in the context of bringing the NPV to zero (**a**) and maintaining maximum economic performance (**b**).

In the context where the aim is to achieve an NPV of zero, the remuneration for the CO_2_ avoided starts from point 5 on the curve, as shown in Figure 14a, showing that the previous configurations are considered viable from an economic perspective. When there is no heat integration, the amount needed to compensate for each additional ton of CO_2_ avoided is consistently higher than in the scenario with integration. This difference is directly related to the role assigned to bagasse within the biorefinery. While in the first case, part of the bagasse is always used as fuel, the second scenario maximizes its use, using it both to generate heat and to produce methanol. This integrated approach not only increases the amount of CO_2_ avoided but also reduces the amount required per ton to balance the economic costs of the system. The compensation cost per ton of CO_2_ avoided, which is necessary to maintain maximum economic performance, remains higher in the scenarios without energy integration, reflecting the reasons previously discussed. When comparing these values with the historical average price of BRL 85.67 [39] paid per CBIO and considering an exchange rate of BRL 5.50 per dollar, it can be seen that the value of USD 15.57 per ton of CO_2_ avoided is relatively close to the values recorded in the first four points of the curve with energy integration, which reach USD 19.81. This suggests that methanol production through bagasse gasification, especially when its surplus heat is properly utilized, is a promising technology to be incorporated into sugarcane biorefineries in the future, corroborating the findings of other authors [40].

## 5. Conclusions

This study proposed an innovative superstructure formulation based on mixed-integer linear programming (MILP) to identify optimal production and configuration strategies for sugarcane biorefineries, applying multi-objective optimization to simultaneously assess economic and environmental performance. The focus was on integrating biomethanol production within a sugarcane biorefinery located in São Paulo, Brazil. Comparing scenarios with and without energy integration, the results clearly indicate superior economic and environmental outcomes when heat integration is applied. The integration enables the transfer of excess heat between processes, allowing bagasse, traditionally directed to cogeneration systems, to be increasingly allocated to the gasification process for methanol production. This shift allows gasification to act as a partial utility system, reducing reliance on cogeneration for steam supply. Consequently, the amount of CO_2_ avoided increases by 19.28% at the environmental extreme, although this is accompanied by a reduction in net present value (NPV). Hydrogen production technologies were also evaluated, with biomethane steam reforming identified as the most economically advantageous option, followed by alkaline water electrolysis powered by photovoltaic electricity. The adoption of electrolysis increases methanol production without additional CO_2_ emissions, further enhancing the biorefinery’s environmental performance. Quantitatively, the biorefinery achieved a maximum energy efficiency of 57.3% when scaling up gasification, but its efficiency decreases when e-methanol production starts. Comparing the cases with and without energy integration, heat recovery was responsible for increasing the system’s energy efficiency by 8% and net energy production by 11%. The total biomethanol production potential reached up to 5.8 Mt via gasification and 3.5 Mt of e-methanol through CO_2_ catalytic hydrogenation. Despite improved environmental performance leading to negative NPV values in several configurations, a CO_2_ avoidance remuneration ranging from USD 3.27 to USD 129.79 per ton could render the process economically feasible. The cost of avoided CO_2_ per ton was lower in integrated energy scenarios, demonstrating the economic advantage of heat recovery. The study highlights the critical trade-off between profitability and sustainability, showing that energy integration and process optimization are key levers to improve environmental metrics without excessively compromising economic viability. Bagasse gasification, especially when coupled with effective heat integration and supported by public policies for carbon credit remuneration, emerges as a promising pathway for future sugarcane biorefineries aiming to balance economic and environmental goals. For future work, the authors believe that other important aspects for the development and optimization of biorefineries could be incorporated into the formulation presented in this work, such as the influence of resource transportation through logistics optimization and parameter uncertainty through stochastic optimization or robust optimization, which are limitations of the presented model. The incorporation of these other aspects could bring new insights into relevant current issues, such as the production of marine biofuels and SAF.

## Figures and Tables

**Table 2:** Values of the objective functions for maximum environmental and economic performance in cases with and without energy integration.

		Maximum Economic Performance	Maximum Environmental Performance
Case 1	OF1 (USD)	6.49 × 10^9^	−4.56 × 10^10^
	OF2 (tonCO_2_eq/year)	1.04 × 10^7^	4.33 × 10^7^
Case 2	OF1 (USD)	6.49 × 10^9^	−5.98 × 10^10^
	OF2 (tonCO_2_eq/year)	1.04 × 10^7^	3.63 × 10^7^

**Table 3:** Maximum performance indicator values found for the case with and without heat integration.

	Energy Efficiency (%)	Net Energy Production (MJ/Year)	Net Energy Production per Area (GJ/Year.ha)
With Heat Integration	57.3	2.5 × 10^11^	322.47
Without Heat Integration	52.4	2.34 × 10^11^	288.96

## Data Availability

The original contributions presented in the study are included in the article/Supplementary Materials. Further inquiries can be directed to the corresponding author.

## Supplementary Materials

The following supporting information can be downloaded at https://www.mdpi.com/article/10.3390/e27111162/s1. Figure S1. Identification of the thermally integrated configuration that offers maximum environmental performance without energy integration; Figure S2. Analysis of the composition of costs related to avoided CO_2_ in various scenarios generated along the Pareto curve; Table S1. Linearized cost curve coefficients and their respective levels; Table S2. Heat streams considered for each process; Table S3. Purchase price (RMP) and sale price (MP) of the main resources considered; Table S4. Amount of CO_2_eq emitted (inCO_2_) by the resource and its fossil equivalent of the main resources considered; Table S5. Processes that compose the biorefinery in the configurations obtained, for the case with heat integration; Table S6. Processes that compose the biorefinery in the configurations obtained, for the case without heat integration; Table S7. Amount produced of each resource by period for the cases studied. References [41,42,43,44,45] are citied in the Supplementary Materials.
