# Supplementary material for: A Multi-Objective Framework for Biomethanol Process Integration in Sugarcane Biorefineries Under a Multiperiod MILP Superstructure
Source: Entropy (Basel). 2025 Nov 15;27(11):1162. doi: 10.3390/e27111162 (PMC12651205; doi:10.3390/e27111162)
Supplement: Supplementary file 1 [file entropy-27-01162-s001.zip › entropy-3953234-Supplementary.pdf]

SUPPLEMENTARY MATERIAL

A Multiobjective Framework for Biomethanol Process Integration in Sugarcane Biorefineries under Multiperiod MILP Superstructure

Victor Fernandes Garcia [1], Reynaldo Palácios[2] and Adriano Viana Ensinas [3,*]

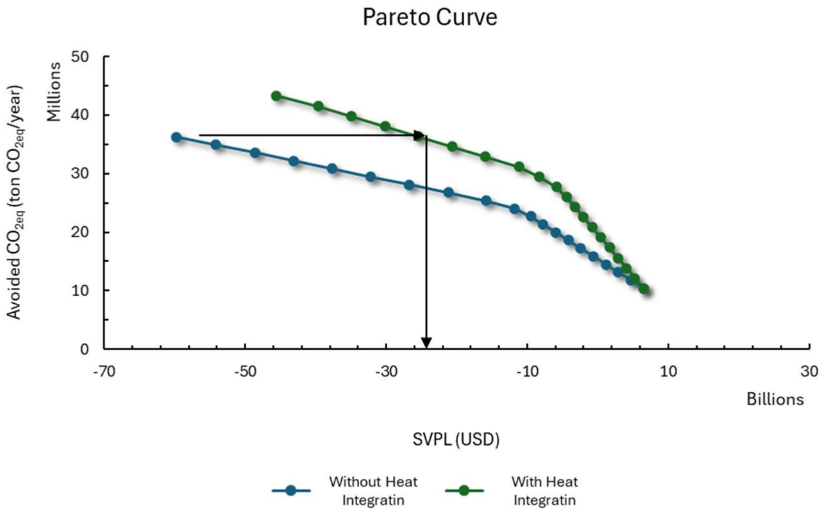

Figure S1 – Identification of the thermally integrated configuration that offers maximum environmental performance without energy integration.

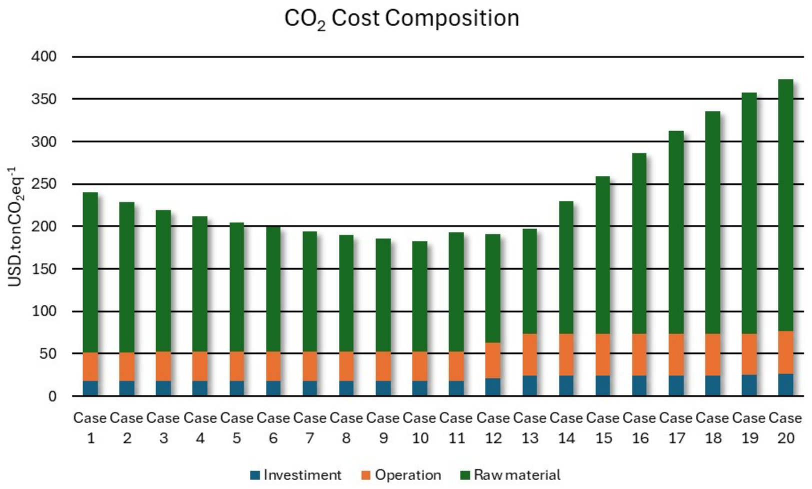

Figure S2 - Analysis of the composition of costs related to avoided CO_2 in various scenarios generated along the Pareto curve.

Table S1 - Linearized cost curve coefficients and their respective levels.

Unit	Level	CapMax	CapMin	capCostA	capCostB
Dest1G	L1	0.35	0.1	18.21	124.83
Dest1G	L2	1.4	0.35	41.13	73.37
Dest1G	L3	2	1.4	63.98	56.04
Dest1G	L4	100	2	91.9962	0
Bag2MeOH	L1	0.72	0.2	499.20	91.14
Bag2MeOH	L2	1	0.72	409.13	151.58
Bag2MeOH	L3	100	1	560.71	0.00
Cogen9	L1	0.35	0.1	5.22	35.80
Cogen9	L2	1.4	0.35	11.80	21.04
Cogen9	L3	1.5	1.4	16.80	17.08
Cogen9	L4	100	1.5	30.20	0
Cogen6	L1	0.35	0.1	5.22	35.80
Cogen6	L2	1.4	0.35	11.80	21.04
Cogen6	L3	1.5	1.4	16.80	17.08
Cogen6	L4	100	1.5	30.20	0
Cogen2	L1	0.35	0.1	5.22	35.80
Cogen2	L2	1.4	0.35	11.80	21.04
Cogen2	L3	1.5	1.4	16.80	17.08
Cogen2	L4	100	1.5	30.20	0
Bag2EEL	L1	0.35	0.1	7.98	54.74
Bag2EEL	L2	1.4	0.35	18.04	32.17
Bag2EEL	L3	2.7	1.4	30.94	22.91
Bag2EEL	L4	100	2.7	41.02	
H2PEM	L1	0.35	0.1	11.393	78.113
H2PEM	L2	1	0.35	22.465	50.852
H2PEM	L3	100000	1	73.317	0
CO2MeOH	L1	0.35	0.1	34.5	5.03
CO2MeOH	L2	1.4	0.35	20.28	11.37
CO2MeOH	L3	1.5	1.4	16.46	16.18
CO2MeOH	L4	100	1.5	27.25	0
Vin2CH4	L1	0.35	0.1	1.99	13.62
Vin2CH4	L2	1.4	0.35	4.49	8.01
Vin2CH4	L3	1.5	1.4	6.39	6.50
Vin2CH4	L4	100	1.5	10.72	0
EtOH_SMR	L1	0.35	0.1	3.88	0.46
EtOH_SMR	L2	1.4	0.35	2.44	1.10
EtOH_SMR	L3	1.5	1.4	2.03	1.62
EtOH_SMR	L4	1000	1.5	3.11	0.00
SMR	L1	0.35	0.1	3.08	0.36
SMR	L2	1.4	0.35	1.93	0.87
SMR	L3	1.5	1.4	1.61	1.28
SMR	L4	1000	1.5	2.47	0.00

FC2CH4	L1	0.35	0.1	11.34	77.78
FC2CH4	L2	1.4	0.35	25.63	45.71
FC2CH4	L3	1.5	1.4	36.49	37.11
FC2CH4	L4	100	1.5	61.22	0.00
Boiler LP	L1	0.72	0.2	12.6406	2.3078
Boiler LP	L2	1	0.72	10.3599	3.8382
Boiler LP	L3	100	1	14.1981	0
Boiler MP	L1	0.72	0.2	12.6406	2.3078
Boiler MP	L2	1	0.72	10.3599	3.8382
Boiler MP	L3	100	1	14.1981	0

Dest1G – Destillery; Bag2MeOH – Bagasse Gasification; Cogen9 – Cogeneration system steam 9bar; Cogen6 – Cogeneration system steam 6 bar; Cogen2 – Cogeneration System; Bag2EEL – Bagasse Power Plant; H2PEM – Hydrogen Proton Exchange Membrane; CO2MeOH – CO2 Catalytic Hydrogenation; Vin2CH4 – Vinasse Biodigestion; EtOH_SMR – Ethanol Steam Reforming; SMR – Stem Methane Reforming; FC2CH4 – Filter cake biodigestion;

Table S2 - Heat streams considered for each process.

Unit	Nstream	Tin [K]	Tout [K]	dH [MW]
Distillery	1	403.15	305.15	41.00
Distillery	2	305.15	301.15	12.20
Distillery	3	377.05	308.15	3.00
Distillery	4	382.45	308.15	37.20
Distillery	5	351.45	308.15	8.60
Distillery	6	383.15	308.15	8.40
Distillery	7	358.05	308.14	19.50
Distillery	8	354.85	354.84	26.40
Distillery	9	351.45	351.44	7.40
Distillery	10	298.15	323.15	4.40
Distillery	11	307.35	378.15	44.00
Distillery	12	371.25	388.15	2.70
Distillery	13	368.65	403.15	14.60
Distillery	14	304.35	363.15	33.70
Distillery	15	382.45	382.46	43.70
Distillery	16	287.05	377.06	21.80
Distillery	17	407.65	407.66	6.70
Distillery	18	422.75	422.76	2.50
Bag2MeOH	1	351.5	498.2	17.3
Bag2MeOH	2	523.2	366.5	22.2
Bag2MeOH	3	366.5	316.5	12.7
Bag2MeOH	4	394.0	394.2	4.9
Bag2MeOH	5	395.9	335.9	0.2
Bag2MeOH	6	1,183.2	354.8	68.2
Bag2MeOH	7	468.7	354.8	6.0
Bag2MeOH	8	456.3	354.8	5.1
Bag2MeOH	9	298.0	438.2	4.2
Bag2MeOH	10	438.2	439.2	14.6
Bag2MeOH	11	439.2	1,144.3	11.0
Bag2MeOH	12	298.2	400.0	3.9
Bag2MeOH	13	400.0	401.0	20.1
Bag2MeOH	14	401.0	533.2	2.5

Bag2MeOH	15	513.15	512.15	53
CO2MeOH	1	410.15	318.15	1.44
CO2MeOH	2	430.15	318.15	1.80
CO2MeOH	3	431.15	318.15	1.96
CO2MeOH	4	530.15	308.15	7.16
CO2MeOH	5	364.15	493.15	24.11
CO2MeOH	6	508.15	313.15	66.66
CO2MeOH	7	390.15	308.15	14.53
CO2MeOH	8	393.35	390.65	17.70
CO2MeOH	9	426.05	433.95	37.41
EtOH_SMR	1	297.59	623.15	3.88
EtOH_SMR	2	1,119.15	873.15	0.93
EtOH_SMR	3	673.55	513.65	0.56
EtOH_SMR	4	393.45	654.45	1.91
EtOH_SMR	5	1,222.25	382.95	7.56

Bag2MeOH: Bagasse Gasification; CO2MeOH: Catalytic CO2 Hydrogenation; EtOH_SMR: Ethanol Steam Reforming;

Table S3 - Purchase price (RMP) and sale price (MP) of the main resources considered.

Resource	RMP(USD/ton)	MP(USD/ton)	
Sugarcane	23.00	-	
Electricity	70.00	70.00	[1]
Ethanol	674.27	674.27	[2]
Bagasse	-	-	
MeOH	350.00	350.00	[3]
CO_2	-	-	
Vinasse	-	-	
CH_4	568.28	568.28	[4]
FilterCake	-	-	
Fotovoltaic Electricity	70.00	70.00	[1]

Table S4 – Amount of CO2eq emitted (inCO2) by the resource and its fossil equivalent of the main resources considered.

Resource	emCO$_2$ (ton CO$_2$eq/ton)	avCO$_2$ (ton CO$_2$eq/ton)	Reference
Sugarcane	0.065	N.A.	[5]
Electricity	0.03	0.03	[6]
Ethanol	N.A.	2.64	[5]
MeOH	N.A.	3.50	[5]
CH_4	N.A.	2.80	[5]
FilterCake	N.A.	N.A.	[5]
Fotovoltaic Electricity	0	N.A.	
MeOH to exportation	N.A.	2.80	[5]

Table S5 - Processes that compose the biorefinery in the configurations obtained. for the case with heat integration

Configuration	Main process
C1	SD + BP + VBD + Cogeneration 9 – 6 – 2 bar
C2	SD + BP + BG + VBD + Cogeneration 6 – 2 bar
C3	SD + BG + VBD + SMR + CCH + Cogeneration 9 – 6 – 2 bar
C4	SD + BG + VBD + SMR + CCH + FCBD + E-PEM
C5	SD + BG + VBD + CCH + FCBD + E-PEM

SD – Sugarcane Destilery; VBD – Vinasse Biodigestion; FCBD – Filtercake Biodigestion; BG - Bagasse Gasification; CCH – Catalytic CO2 Hydrogenation; E-PEM – Electrolysis PEM; SMR – Steam Methane Reforming

Table S6 - Processes that compose the biorefinery in the configurations obtained. for the case without heat integration.

Configuration	Main Processes
C1’	SD + BP + VBD + Cogeneration 9 – 6 – 2 bar
C2’	SD + BP + VBD + BG + Cogeneration 9 – 6 – 2 bar
C3’	SD + VBD + FCBD + BG + CCH + E-PEM + SMR + Cogeneration 9 bar + Boiler 6 bar
C4’	SD + VBD + FCBD + BG + CCH + E-PEM + Cogeneration 9 bar + Boiler 6 bar

SD – Sugarcane Destilery; VBD – Vinasse Biodigestion; FCBD – Filtercake Biodigestion; BG - Bagasse Gasification; CCH – Catalytic CO2 Hydrogenation; E-PEM – Electrolysis PEM; SMR – Steam Methane Reforming

Table S7 - Amount produced of each resource by period for the cases studied.

Resources	Period	C1	C2	C3	C4	C5
Ethanol	1	5,691,256	5,691,256	5,691,256	5,691,256	5,691,256
Ethanol	2	-	-		-	-
Biomethane	1	235,380	235,380	235,380	241,072	288,361
Biomethane	2	-	-	-	3,751	34,919
Electricity	1	10,373,605	9,457,233	1,297,229	-	-
Electricity	2	4,548,440	4,079,287	-	-	-
Bio/e-Methanol	1	-	304,980	3,185,908	5,894,753	7,111,050
Bio/e-Methanol	2	-	201,009	1,976,666	841,933	2,334,696

Note: Period 1 = Season; Period 2 = off Season;

REFERENCES

1. NATIONAL ELECTRIC AGENCY (ANEEL). Database of Electricity Distribution Tariffs. . [s. d.]. Available at: https://portalrelatorios.aneel.gov.br/luznatarifa/basestarifas
2. BRAZIL. MINISTRY OF SCIENCE. TECHNOLOGY AND INNOVATION. SIRENE – National Emissions Registry System.. [s. d.]. Available at: https://www.gov.br/mcti/pt-br/acompanhe-o-mcti/sirene/dados-e-ferramentas. Accessed on May 13. 2024.
3. CENTER OF ADVANCED STUDIES ON APPLIED ECONOMICS (CEPEA). Ethanol | Monthly Indicator of Hydrous Fuel Ethanol CEPEA/ESALQ - São Paulo. . [s. d.]. Disponível em: https://www.cepea.org.br/br/consultas-ao-banco-de-dados-do-site.aspx. Accessed on May 1. 2024.
4. INSTITUTO 17. Biogas in Brazil: economic feasibility analysis and investment potential. Brazil Energy Programme – BEP (Brazil). Technical report 02-2022. São Paulo. SP: Instituto 17. 2022.
5. KANG. S.; BOSHELL. F.; GOEPPERT. A.; PRAKASH. S. G.; LANDÄLV. I.; SAYGIN. D. Innovation outlook: renewable methanol. Abu Dhabi: International Renewable Energy Agency. 2021.
6. MATSUURA. M. I. da S. F.; SEABRA. J. E. A.; CHAGAS. M. F.; SCACHETTI. M. T.; MORANDI. M. A. B.; MOREIRA. M. M. R.; NOVAES. R. M. L.; RAMOS. N. P.; CAVALETT. O.; BONOMI. A. RenovaCalc: The RenovaBio Program Calculator. In Proceedings of the Embrapa Meio Ambiente Conference. Jaguariúna. Brazil. 2018; pp. 162-167.
